# Mid-Infrared Gas Sensing Based on Electromagnetically Induced Transparency in Coupled Plasmonic Resonators

**DOI:** 10.3390/s23229220

**Published:** 2023-11-16

**Authors:** Sarah Shafaay, Sherif Mohamed, Mohamed Swillam

**Affiliations:** Department of Physics, School of Sciences and Engineering, The American University in Cairo, New Cairo 11835, Egypt; sara.shafaay@aucegypt.edu (S.S.); sherifms@aucegypt.edu (S.M.)

**Keywords:** coupled-ring resonators, plasmonic mode, mid-infrared spectral range, doped silicon, optical sensors

## Abstract

The existence of surface plasmon polaritons in doped silicon micro-scale structures has opened up new and innovative possibilities for applications, such as sensing, imaging, and photonics. A CMOS-compatible doped Si plasmonic sensor is proposed and investigated. The plasmon resonance can be tuned by controlling the carrier density and dopant concentration. In this paper, we demonstrate that using silicon doped with phosphorus at a concentration of 5 × 10${}^{20}$ cm${}^{-3}$ can induce surface plasmon resonance in the mid-infrared region. Two ring resonators of two different radii based on metal–insulator–metal waveguide structures are studied individually. Then, the two ring resonators are integrated in the same device. When the two ring resonators are coupled and resonate at the same frequency; two distinct resonance spectral lines are generated with striking features that improve its potential use for sensing and modulation applications. The propagating plasmonic mode is studied, including its mode profile and bend loss. We evaluate the effectiveness of a microstructure gas sensor with dimensions of 15 μm × 15 μm by measuring its sensitivity and selectivity towards methane and ethane gases. Small alterations in the surrounding refractive index led to noticeable shifts in the resonance peak. The sensor achieved a sensitivity of 7539.9 nm/RIU at the mid-infrared spectral range around the 7.7 μm wavelength. Furthermore, by combining the resonators, we can achieve a smaller full width at half maximum (FWHM), which will ultimately result in greater sensitivity than using a single-ring resonator or other plasmonic resonator configurations. Once the sensitivity and selectivity of the sensor are measured, the FOM can be calculated by dividing the sensitivity by the selectivity of the sensor, resulting in an FOM of 6732.

## 1. Introduction

The silicon-on-insulator (SOI) platform is the most commonly used platform in the photonics industry owing to the strong waveguiding properties in the high-index silicon Si compared to the low-index silicon dioxide SiO${}_{2}$ [1,2,3]. In addition, SOI is characterized by its CMOS compatibility and fabrication feasibility [4,5]. Thus, the SOI platform is extensively investigated for various photonic components with different geometries, such as gratings, waveguide dividers and combiners, multimode interferometers, directional couplers, and ring resonators. These components are used for various applications [6,7,8]. In particular, for sensing applications, the challenge of maximizing light interaction with the analyte arises in the SOI platform as a result of the small overlap between the analyte and the decaying exponential tail of the waveguide mode in the clad. So, slot waveguides were introduced to overcome such a challenge, where light confined in the slot interacts strongly with the analyte that physically fills the same slot. Moreover, Si is transparent in the near-infrared spectral region that possesses the important 1.55 μm wavelength, which is the fundamental wavelength in the telecommunication industry and optical fibers data transmission [9,10]. In general, one of the advantages of a doped silicon platform is that it allows for the control of doping. However, plasmonic resonators, in general, do not have a small full width at half maximum (FWHM), so they are not very good candidates for sensing because of the low sensitivity due to the increased amount of losses that definitely increase the FWHM. Accordingly, we are trying to increase the sensitivity of the plasmonic sensor by using a coupled-ring resonator, where the FWHM in the combined system is smaller, and this will increase the sensitivity compared to a single-ring resonator or other types of resonators using a plasmonic configuration. It also adds the advantage of allowing us to detect more than one gas at the same time by adjusting the split resonance to different gases that can be used simultaneously for more than one gas. We are trying to optimize the design to work around the peaks of methane and ethane, which are important gases in the oil and gas industry and field, and precise measurement is required.

Gas sensing is essential in the detection of toxic greenhouse gases and hazardous inert gases that cause unstable environmental conditions [11,12]. Consistently, gas sensors are essential in various industrial, medical, aerospace, agricultural, and security applications to ensure safe working environments and air quality control [13,14]. Moreover, gas sensors can be used to detect some medical disorders by testing patients’ exhaled breath [15]. The mid-infrared range is of great interest in gas sensing applications, since the absorption peaks of many gases lie within this range [16,17,18,19]. A doped Si platform can be used to offer waveguiding at mid-infrared wavelengths through plasmonic slots. The transparency range of SOI wafers is limited by strong absorption peaks in the mid-infrared (MIR) due to the buried oxide (BOX) layer [20,21]. To overcome the spectral limitations caused by absorption losses, a solution involves using high-quality SOI wafers and locally removing the BOX layer beneath the waveguide. This can be achieved by implementing a suspended membrane-doped silicon waveguidestructure [19,22,23]. An important advantage of doped Si is the plasmonic resonance tunability with doping levels. Controlling the charge density can allow for precise control to the spectral peak of the SPR. Analyzing micron-scale structures using the Drude model shows that the frequency of the SPR varies depending on the dopant concentration. For conventional doping levels (∼10${}^{18}$–10${}^{19}$ cm${}^{-3}$), the SPR frequency is in the far-infrared (FIR) range. However, for heavily doped semiconductors (∼10${}^{20}$ cm${}^{-3}$), the SPR frequency shifts to the mid-infrared (MIR) range, and it can even reach the near-infrared (NIR) range if higher dopant concentrations are achieved (>10${}^{21}$ cm${}^{-3}$). Out of equilibrium methods, such as nanosecond Laser Thermal Annealing (LTA) treatments, can be used to achieve high dopant concentrations in silicon. Through LTA, active phosphorus concentrations as high as 5 × 10${}^{20}$ cm${}^{-3}$ and 2 × 10${}^{21}$ cm${}^{-3}$ can be reached in thin SOI and bulk silicon, respectively [24]. High constraintup to 6.5 × 10${}^{20}$ cm${}^{-3}$ by n-type dopants in Si has been experimentally achieved, as shown in [25]. The field of plasmonics offers promising opportunities for generating, manipulating, and detecting signals using various processes such as generation, processing, transmission, and sensing. These processes can operate at optical frequencies and have potential applications in various fields, including optical communications, biophotonics, sensing, chemistry, and medicine [26], as well as other areas such as broad band metamaterial absorber and versatile full-color nanopainting technology [27,28,29]. Ring resonators are common devices in the photonic domain, and they are used in different applications such as sensors, modulators, and filters [30,31]. The ring resonator is characterized by its discrete sharp resonances and fabrication feasibility [32]. However, when two resonators are coupled in a way that they both resonate at close frequencies, different interesting phenomena can be observed in the whole resonant system. For example, the Fano-resonance occurs between wide and sharper spectral line shapes, resulting in a sharp resonance with a distinct profile [33,34] and electromagnetically induced transparency (EIT), which is the result of differently damped resonators, resulting in a narrow transparency window in the spectrum [35]. Also, the Bormann effect, which is the spatial analog of the EIT effect, is observed in periodic structures such as photonic crystals [34,36].The physical mechanism behind mode splitting in EIT can be understood in terms of the interference between different energy pathways. The propagating light field can interact with multiple resonant modes of the ring resonators, resulting in a superposition of the different energy pathways. This interference can lead to the separation of a single resonance peak into two peaks. The mutual coupling can be adjusted to achieve maximum mode splitting and improved notch depth [37,38]. The EIT effect is attracting special attention as its properties have proved to be useful in different applications, such as quantum information [39], sensitivity filtering, sensing, modulation [35,40,41] optical delay, slow light [42], non-linearity enhancement, and [43] precise spectroscopy [44].

In general, the plasmonic-based resonator suffers from the low-quality factory (Wide FWHM) due to the high intrinsic losses associated with this resonator [45,46]. Hence, using such a resonator for sensing is limited due to its low selectivity and low figure of merit (FOM). In this work, we propose a novel coupled-resonator configuration that increases sensitivity based on splitting the resonance frequency in a coupled-resonance system. This results in a sharper resonance peak of the splitted eigen frequency and hence increases the sensitivity of the combined system.

In this paper, we propose a CMOS-compatible gas sensor based on Si platform based on a coupled-ring resonator. The proposed system utilizes a plasmonic slot configuration for guiding the light using doped silicon materials. First, we study the performance of a single-slotted ring resonator and optimize its performance in the MIR range. We also studied the performance of two coupled resonators by introducing a second resonator designed to work at the same resonance frequency but with a different radius. The coupled resonance system is further tuned to ensure the resonance frequency of both resonators coincides with each other, which results in eigen frequency splitting [47]. The two coupled-ring resonators generate the EIT effect at the resonance frequency of the single resonator. The proposed system produces a sharper resonance peak, and hence its potential for gas sensing in the MIR is studied for different gases. The proposed coupled resonator sensor showed an increased figure of merit (FOM) compared to a single-ring resonator. The application of the proposed system for simultaneous sensing of different gases is also proposed, as is the application of the proposed system for sensing methane and ethane.

## 2. Device Structure

**Figure 1:**
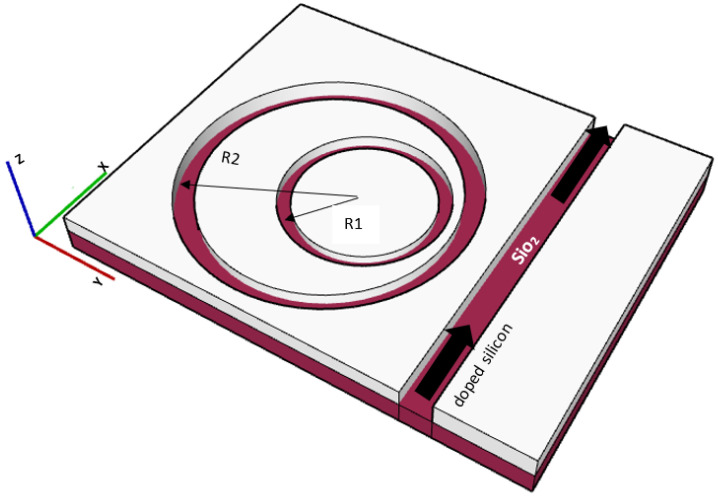
Non-concentric ring resonator device structure.

The proposed sensor is formed of a doped Si wafer on a silicon dioxide, “SiO${}_{2}$”, substrate, and the wafer is etched to form two suspended non-concentric ring resonators. The larger ring has a radius R1, while the smaller ring has a radius R2. The Si wafer is n-doped with phosphorus to tune its plasma resonance to 7.69 μm to enable working in the MIR range. Figure 1 shows the non-concentric ring design where the spacing between the Si slot bus waveguide and the larger ring is equal to the spacing between the two rings.

The complex p1ermittivity of the doped Si is described by the Drude model [48,49,50,51] for metals and is provided by

(1) $$\varepsilon_{m}\left(\omega \right)=\varepsilon_{\infty }-\frac{\omega_{p}^{2}}{\left(\omega^{2}+j\omega \Gamma \right)}$$

where ω is the light frequency in rad/s, $\varepsilon_{\infty }$ = 11.7 F/m is the permittivity at very high frequencies, and $\omega_{p}$ is the plasma frequency, which is provided by

(2) $$\omega_{p}=\sqrt{(}\frac{N_{d}\;q^{2}}{\varepsilon_{0}\;m^{*}})$$

N${}_{d}$ is the free carrier concentration, and $\varepsilon_{0}$ is the free-space permittivity, where the collision frequency israd/s, which is defined as

(3) $$\Gamma =\frac{q}{m^{*}\mu }$$

where $m^{*}$ is the electron effective mass, μ is the carrier mobility, and *q* is the charge of the electron. In order to work in the near-infrared range, which has spectral footprints for different gases, a doping level of 5 × 10${}^{20}$ cm${}^{-3}$ is used. The Drude model parameters of ω${}_{p}$ = 2.47 × 10${}^{15}$ rad/s and Γ = 9.4 × 10${}^{9}$ rad/s describe such doped Si material [1]. Our model has been tested and showed good agreement with the empirical model and the experimental measurements by Palik [52] for various concentrations up to 10${}^{20}$ cm${}^{-3}$.

## 3. Ring Resonator Characterization

### 3.1. Plasmonic Mode Analysis

**Figure 2:**
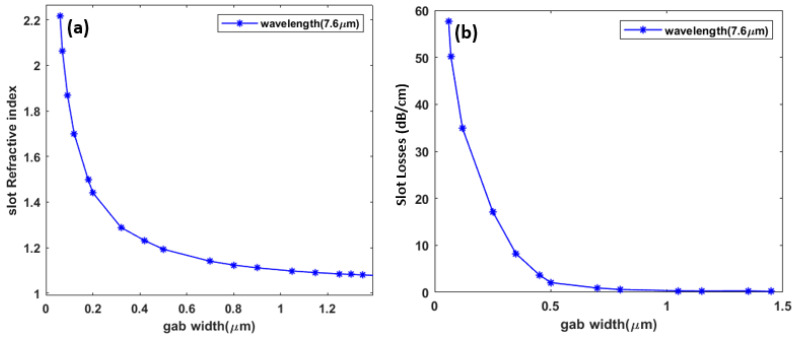
Metal–insulator–metal waveguide with height 1 μm. (**a**) shows the relation between the slot gap width and the effective refractive index at 7.6 μm; (**b**) shows the relation between the slot gap width and mode loss at 7.6 μm.

When the bus plasmonic waveguide is excited by a TE polarized source, a plasmonic mode starts to propagate in the 1 μm wide metal–insulator–metal waveguide until it couples to the ring structure which is itself a metal–insulator–metal type waveguide of the same width of 1 μm. The relationship between slot width, effective refractive index, and losses is important for optimizing waveguide loss. As shown in Figure 2, the mode experiences its minimum loss at around 1 μm. One of the main advantage of working in the MIR is the relaxed constraints in the dimensions, which provide an ease of fabricating plasmonic devices. This is mainly due to the dimensions of the guided wavelengths, which are in the order of a few microns. For example, our proposed plasmonic slot waveguide is a single mode up to 1.7 μm. Therefore, a slot width of 1 μm is a good choice because it is both easy to fabricate and has minimal loss. Additionally, increasing the width provides the ability to operate further away from the plasmon wavelength.

**Figure 3:**
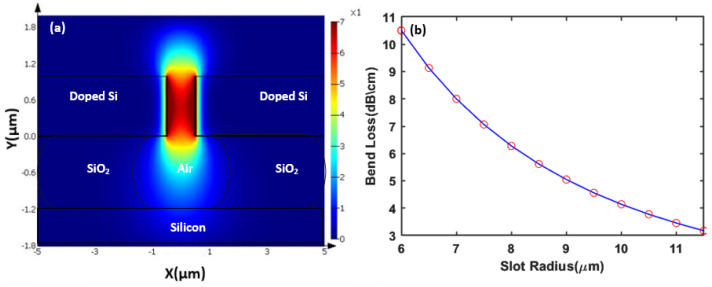
Metal–insulator–metal waveguide. (**a**) real |Hy| mode profile; (**b**) bend loss.

Moreover, the mode profile and the bend loss of the induced plasmonic mode are studied in Figure 3, where a waveguide width of 1 μm has a complex index of 1.00049 + 3.195614 × 10${}^{-3}$ i and 8.2033 dB/cm modal loss at 7.7 μm wavelength. A waveguide simulator is used for the modal analysis and bend loss calculations [53]. The bend loss of our Si slot waveguide shown in Figure 3b is smaller than the modal loss; this allows us to minimize the rings radii to achieve a compact design with a small footprint.

### 3.2. Single-Ring Resonator Behavior

**Figure 4:**
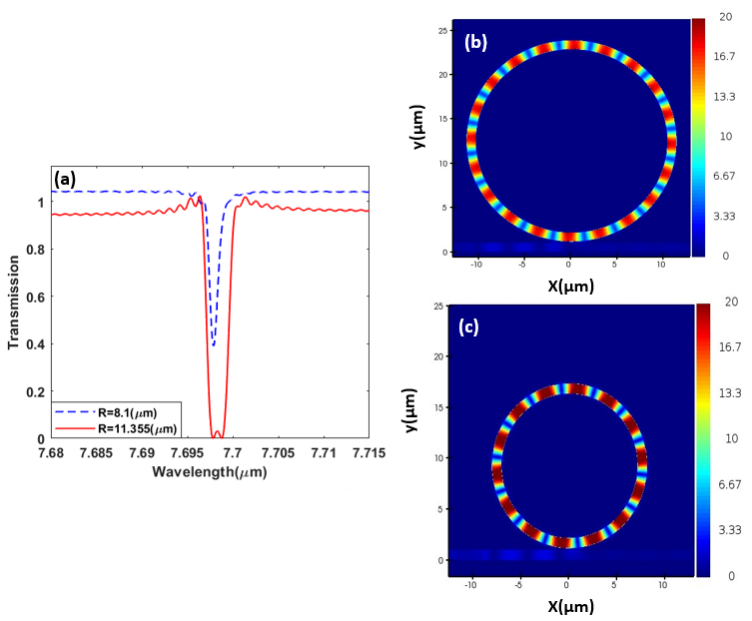
Single—ring resonator transmission spectrum (**a**). Electric field distribution |Ex| at the resonance 7.69 μm wavelength for (**b**) R = 8.1 μm and (**c**) R = 11.355 μm, with each simulated separately.

The electric field (E-field) can be confined in a plasmonic ring resonator through the phenomenon of plasmonics, which involves the interaction between light and free electrons in metal structures. Specifically, in a plasmonic ring resonator, the interaction between an electromagnetic wave and a circular metallic structure causes the confinement of the electric field within the cavity region [54]. By adjusting the dimensions and material properties of the plasmonic ring resonator, the wavelength and intensity of the confined E-field can be precisely controlled. Hereby, we study the behavior of the single-ring structure. The excited ring resonator has its resonance positions described by the optical path lengths of the different resonance orders, which are given by

(4) $$\lambda_{m}=\frac{2\pi \;R\;n_{eff}}{m}$$

where $n_{eff}$ is the effective index, and m is an integer representing the resonance order. Figure 4 shows the spectrum measured at the through port of the bus waveguide of two rings with different radii where we simulate each ring separately and optimize its radius so both rings resonate at the same frequency/wavelength and can be coupled in a design integrating both rings. Hereby, the two rings have a resonance line at 7.69 μm in the mid-infrared range. A 2D electromagnetic simulator is used for the design and analysis of our structures [55]. The silicon slot can be coupled using conventional methods of coupling from silicon to plasmonic slots configuration [56,57].

### 3.3. Coupled-Ring Resonator Behavior

**Figure 5:**
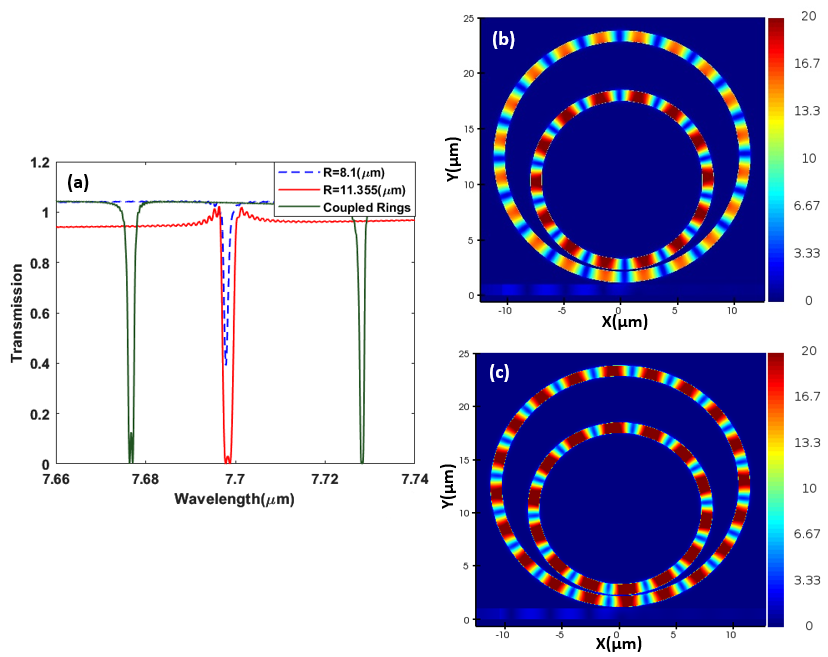
Coupled—ring resonator transmission spectrum in comparison to the single resonator spectrum. (**a**) Coupled—ringresonator electric field distribution |Ex| at (**b**) λ = 7.67 μm and (**c**) λ = 7.72 μm.

The objective, here, is to design two rings of different radii that can be made compact by figuring out an appropriate geometrical arrangement. However, when two resonators are coupled and optimized to resonate at the same or very close frequencies, interesting effects could be observed, such as the Fano resonance and electromagnetically induced transparency effects. When a tunable TE source is introduced into the bus waveguide, it couples with the field of the two slot ring resonators, causing the light field’s propagation to be affected. Specifically, the interaction between the propagating light field and the resonant modes of the ring resonators results in a change in the refractive index of the waveguide. This change in refractive index leads to an alteration of the group velocity of the light field, which can be controlled by adjusting the coupling strength between the waveguide and the resonators. In other words, it is reasonable to expect that if we have two resonances of equal frequency, the eigen state will split into two eigen states [47]. Correspondingly, the EIT is shown in Figure 5, where we observe an absence of the resonant spectral lines of the same frequency/wavelength but the generation of two resonance lines at red-shifted and blue-shifted frequencies, where the mode splitting occurs due to the interference between various energy pathways. In other words, when optical resonators create optical transparency inside the absorption window through coherent interference between the resonating modes This effect is known as the EIT effect.

Initially, for only a single ring, each of the two rings was optimized to resonate around a 7.69 μm wavelength, as observed in Figure 5, in agreement with (5). However, when the two rings are coupled, the EIT effect causes the initial resonances at 7.69 μm to disappear, and two new far apart resonances at the 7.67 μm and the 7.72 μm wavelengths are generated.

## 4. Ring Resonator Sensor

**Figure 6:**
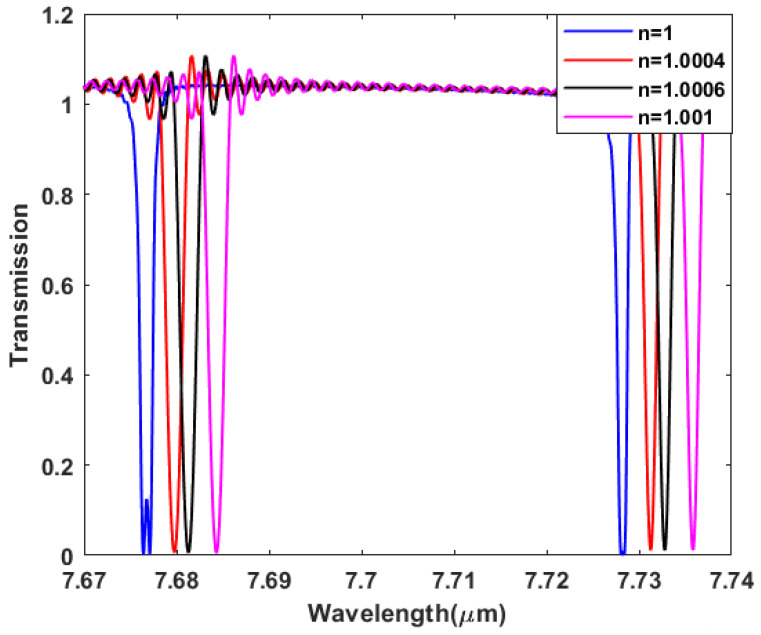
Spectral resonance red shift with surrounding gas index.

**Figure 7:**
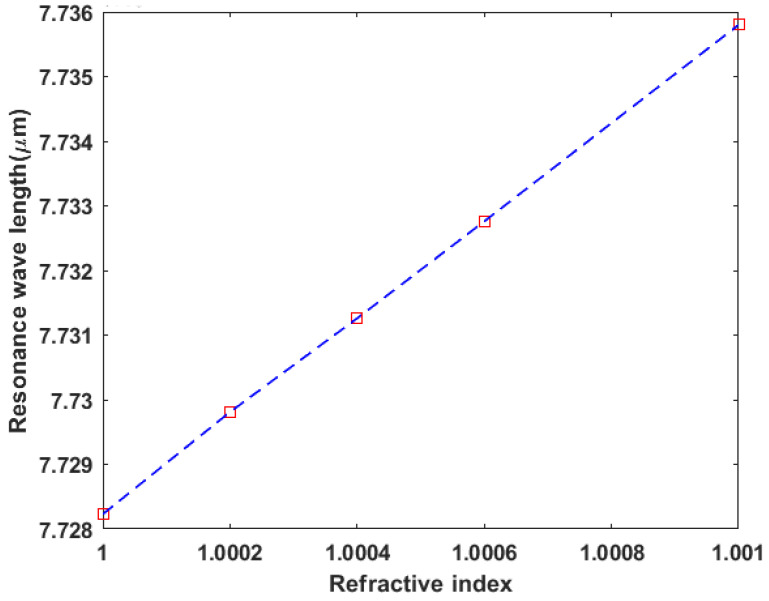
Resonance wavelength position vs. surrounding gas index.

We can make use of the generated EIT effect and the sharp resonances of the proposed coupled-ring resonator structure in sensing applications. When the structure is subject to the environment, the surrounding gas fills the empty volume of the structure, i.e., the insulator (air) in the metal–insulator–metal bus and ring resonator waveguides mentioned previously. So, changes in the gas mixture of the surrounding environment result in a change in the surrounding gas refractive index which is followed by a change in the plasmonic mode effective index, thereby altering the resonance position and causing a shift in the induced EIT effect spectral resonances. Figure 6 demonstrates the generated EIT spectral resonances at different surrounding refractive indices, as we vary the refractive index from 1 to 1.001 in increments of 0.0002, where the spectral resonances are blue-shifted from 7.728 μm to 7.736 μm as the refractive index increases. Figure 7 illustrates the linear relationship between the shifted wavelength and the change in refractive index.The sensor sensitivity defined by Δλ/Δn is calculated from the slope of Figure 7 to be 7539.9 nm/RIU, while the figure of merit is defined by the sensitivity divided by the full length at half maximum of the resonant line, i.e., FOM = Δλ/(Δn.FWHM).

## 5. Analysis for Testing Hydrocarbon Gases

We study the sensitivity of our plasmonic sensor to detect ethane (C${}_{2}$H${}_{6}$) and methane (CH${}_{4}$) gases. The frequency shift and splitting are also expected when the designed resonator coincides with the resonance frequency of the gas molecules. This is one of the reasons that our proposal works closely with the resonance frequency (absorption peak of the gas) while avoiding coinciding with it. By using the gas absorption data from the National Institute for Standards and Technology “NIST” database [58], both the real and the complex refractive indices were calculated by Equations (5) and (6), where Equation (5) relates the absorption α(λ) to the extinction coefficient *k*(λ):

(5) $$\alpha \left(\lambda \right)=\frac{4\pi k(\lambda )}{\lambda }$$

**Figure 8:**
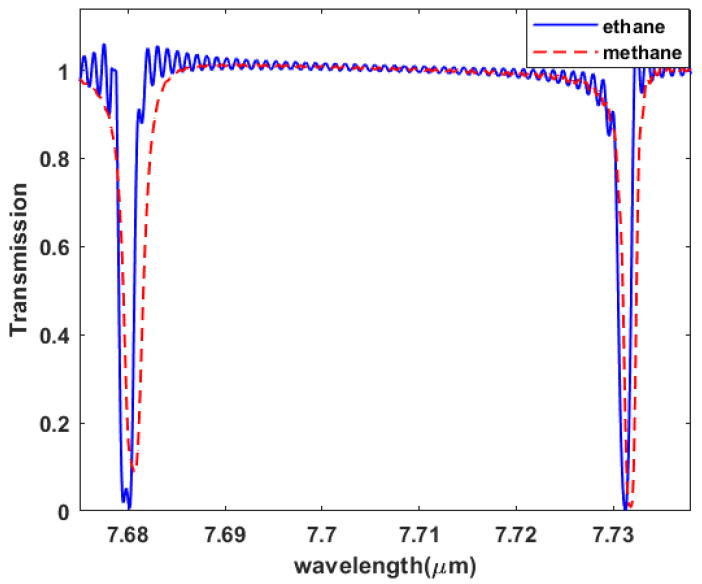
Ring resonance lines in the presence of methane and ethane gases.

Equation (6) is the Kramers–Kronig relation, which allows us to estimate the refractive index (*n*) at each wavelength λ${}_{0}$ from the extinction coefficient *k*(λ) by implementing [59].

(6) $$n\left(\lambda_{0}\right)=n\left(\lambda_{1}\right)+p\frac{2(\lambda_{1}^{2}-\lambda_{0}^{2})}{\pi }\int_{0}^{\infty }\frac{\lambda k(\lambda )d\lambda }{\left(\lambda_{0}^{2}-\lambda^{2}\right)\left(\lambda_{1}^{2}-\lambda^{2}\right)}$$

where *p* is the Cauchy principle value of the integral, and *n*(λ${}_{1}$) is a known refractive index of the gas at wavelength (λ${}_{1}$). Furthermore, we test our sensor to detect and differentiate between methane and ethane gases. Methane (CH${}_{4}$) is an inflammable gas that is devoid of both smell and color. It serves as the primary constituent of natural gas and holds immense importance as a global fuel source for electricity production and heating. However, it also plays a substantial role in contributing to climate change. Methane acts as a potent greenhouse gas (GHG) with a global warming potential 28 times higher than carbon dioxide (CO${}_{2}$) over a span of 100 years [60]. The industrial revolution has led to a substantial rise in atmospheric methane concentration, soaring from approximately 800 parts per billion (ppb) in the early 1900s to over 1800 ppb in 2016 [61]. This increase can be mainly attributed to anthropogenic sources, such as landfills, animal waste management systems, coal mining, petrochemical exploration, power transformers, and oil and gas distribution and production facilities [60]. It is important to note that methane gas is flammable and can become explosive if its concentration reaches 5 to 15% in a confined space [62]. Despite its negative environmental impacts, natural gas remains highly sought after due to its abundance and cleaner combustion process, ensuring its continued widespread use [63]. In fact, natural gas is replacing coal, particularly in the power sector in the United States, due to its lower CO${}_{2}$ emissions during combustion and its lower production costs [64]. Furthermore, projections indicate that natural gas will become the second most prominent energy source in the future [65]. In Figure 8, we show how our sensor is able to detect and differentiate between methane and ethane gases.

**Figure 9:**
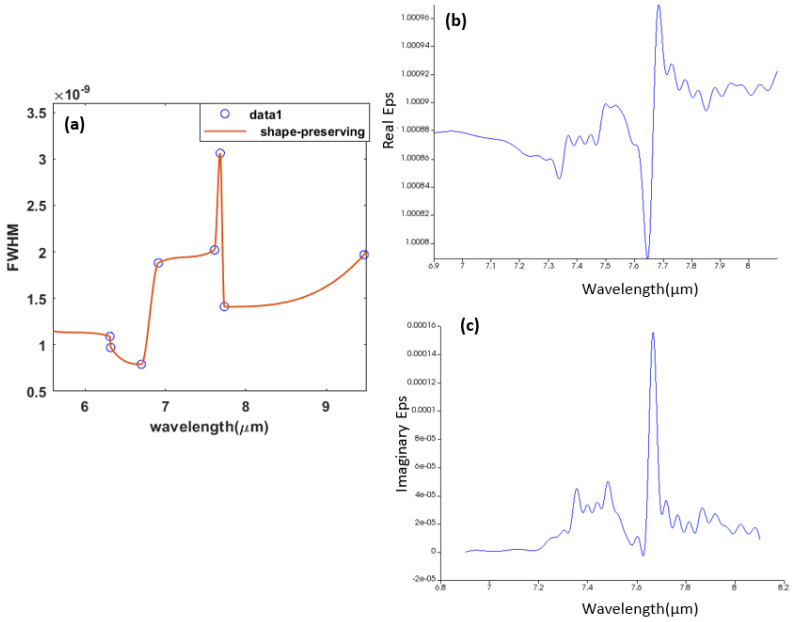
(**a**) shows how the full width at half maximum increases as it gets closer to the absorption peaks; (**b**) shows how the methane real refractive index varies with the wavelength; and (**c**) shows how the methane imaginary refractive index varies with the wavelength.

The sensing process for our sensor depends on detecting the change in the refractive indices, which allows us to differentiate between gases and know whether they are pure gases or mixed gases. For illustration, the blue line shows the presence of pure ethane gas, while the red line shows the presence of pure methane gas. The refractive index changes proportionally with the percentage of the methane–ethane mixture [66], as any percentage change in the methane and ethane mixed gases will cause a shift in the curve. Moreover, to study the sensor selectivity, we found that when working near to the absorption peaks for the interesting gas, the full width at half maximum (FWHM) increases. In this part, we studied the sensor selectivity for methane gas, and we studied the relation between the wavelength and the FWHM near and far from the absorption peaks as shown in Figure 9a. Methane has multiple resonance peaks lying in the region from 6 μm to 9 μm as representing in Figure 9b,c for both real and imaginary eps verses the wavelength respectively.

## 6. Conclusions

A plasmonic electromagnetically induced transparency gas sensor based on doped silicon in the MIR spectral region is designed and optimized. We introduce two non-concentric ring resonators that resonate at the same wavelength to achieve the EIT phenomena. The splitting of resonance frequency in a coupled resonator configuration provides the system with sharp resonance peaks, which in turn leads to an increase in both sensitivity and selectivity. This configuration was tested for detecting ethane and methane gases and achieved a sensitivity of up to 7539.9 nm/RIU and a figure of merit (FOM) of 6231. Additionally, we provide plasmonic mode analysis for the slotted waveguide and the transmission difference between a single-ring resonator and coupled-ring resonator.

## Data Availability

Not applicable.

## Abbreviations

The following abbreviations are used in this manuscript:

SOI	Silicon on Insulator
EIT	Electromagnetically Induced Transparency
FOM	Figure of Merit
MIR	Mid-infrared Range
FWHM	Full Width at Half Maximum
